# Effects of Anodal Cerebellar Transcranial Direct Current Stimulation on Movements in Patients with Cerebellar Ataxias: A Systematic Review

**DOI:** 10.3390/ijerph182010690

**Published:** 2021-10-12

**Authors:** Shu-Mei Wang, Ying-Wa Chan, Yiu-On Tsui, Fong-Yung Chu

**Affiliations:** Department of Rehabilitation Sciences, Faculty of Health and Social Sciences, The Hong Kong Polytechnic University, Kowloon, Hong Kong; cindy1120chan@gmail.com (Y.-W.C.); tsuiyiuon@gmail.com (Y.-O.T.); fannychu222@gmail.com (F.-Y.C.)

**Keywords:** transcranial direct current stimulation, tDCS, cerebellum, ataxia, movement, systematic review

## Abstract

Cerebellar transcranial direct current stimulation (cerebellar tDCS) is a promising therapy for cerebellar ataxias and has attracted increasing attention from researchers and clinicians. A timely systematic review focusing on randomized sham-controlled trials and repeated measures studies is warranted. This study was to systematically review existing evidence regarding effects of anodal cerebellar tDCS on movements in patients with cerebellar ataxias. The searched databases included Web of Science, MEDLINE, PsycINFO, CINAHL, EMBASE, Cochrane Library, and EBSCOhost. Methodological quality of the selected studies was assessed using the Physiotherapy Evidence Database scale. Five studies with 86 patients were identified. Among these, four studies showed positive effects of anodal cerebellar tDCS. Specifically, anodal cerebellar tDCS decreased disease severity and improved finger dexterity and quality of life in patients, but showed incongruent effects on gait control and balance, which may be due to heterogeneity of research participants and choices of measures. The protocols of anodal cerebellar tDCS that improved movements in patients commonly placed the anode over the whole cerebellum and provided ten 2-mA 20-min stimulation sessions. The results may show preliminary evidence that anodal cerebellar tDCS is beneficial to reducing disease severity and improving finger dexterity and quality of life in patients, which lays the groundwork for future studies further examining responses in the cerebello-thalamo-cortical pathway. An increase in sample size, the use of homogeneous patient groups, exploration of the optimal stimulation protocol, and investigation of detailed neural mechanisms are clearly needed in future studies.

## 1. Introduction

Cerebellar ataxias represent a spectrum of neurological disorders resulting from cerebellar abnormalities or a combination of cerebellar and other neurological lesions [1,2,3]. It has been known [4,5] that the cerebellum receives input from multiple cortical areas, including motor areas, and projects back to the source cortical areas via the thalamus, which is called the cortico-cerebello-thalamo-cortical circuitry. Purkinje cells, which are output neurons of the cerebellum [5,6,7], send inhibitory projections to the dentate of the deep cerebellar nuclei to reduce excitatory output from the dentate to the motor cortex so as to affect motor control [6,7,8]. This inhibitory and regulatory effect of the cerebellum on the motor cortex is known as cerebellar inhibition [6,7,8]. The cerebellum plays a significant role in precision and coordination of movements as well as balance [9,10]. Impairments in the cerebellum, Purkinje cells, or cerebellar inhibition are thought to result in cerebellar ataxias [7,9,10]. Indeed, impaired cerebellar inhibition has been shown in patients with cerebellar ataxias [11,12]. Major clinical symptoms of cerebellar ataxias include lack of coordination and balance, dysarthria, dysmetria, tremors, dysdiadochokinesia, and oculomotor deficits [2,13,14]. Cerebellar ataxias may be inherited or sporadic [1,2,3]. The former category encompasses autosomal dominant ataxias (e.g., spinocerebellar ataxias), autosomal recessive ataxias (e.g., Friedreich’s ataxia), mitochondrial ataxias, and X-linked ataxias [1,2,3]. The latter category contains degenerative ataxias (e.g., multiple system atrophy) and acquired ataxias [1,2,3]. It is well known that cerebellar ataxias cause severe physical disabilities and thus wheelchair dependence and significantly reduced quality of life in physical, mental, and social dimensions in patients [2,15,16,17]. Development of effective therapies for these disabling neurological disorders is urgently needed. Nevertheless, heterogeneous manifestations and types of cerebellar ataxias substantially increase difficulty of designing an effective therapy for tackling multiple ataxic symptoms [2].

Notably, in the past decade, cerebellar transcranial direct current stimulation (cerebellar tDCS) has attracted increasing attention from researchers because it directly modulates cerebellar activity and thus shows a high potential for alleviating various symptoms in patients with cerebellar ataxias [16,18,19]. The tDCS is a non-invasive neuromodulation modality that utilizes a weak direct electric current flown from the anode (the positively charged electrode) to the cathode (the negatively charged electrode) to regulate neural excitability and affect human behaviors [6,19,20]. For anodal cerebellar tDCS, the anode is placed over the cerebellum to increase cerebellar excitability and further facilitate movement control; the cathode is put on an irrelevant head/body region [6,19]. As mentioned above, patients with cerebellar ataxias have impaired cerebellar inhibition [11,12]. It is noteworthy that anodal cerebellar tDCS has been found to facilitate cerebellar inhibition in patients with cerebellar ataxias [11,12], as proposed to result from increased excitability of Purkinje cells due to anodal cerebellar tDCS [6,9,11,12]. On the basis of these findings, it can be expected that anodal cerebellar tDCS may be subsequently effective in improving motor control in patients with cerebellar ataxias and could be applied to clinical practice. However, until now, no systematic reviews targeting effects of anodal cerebellar tDCS on patients’ movements have been conducted to gain a comprehensive understanding of the existing evidence. In addition, it remains unclear what protocols of anodal cerebellar tDCS show positive effects on patients’ movements. In addition, a distinct feature of tDCS is provision of the sham/placebo condition, which replicates the electrode placement and the stimulation protocol of the active tDCS condition but provides no electric current stimulation [12]. Randomized controlled trials and repeated measures studies on tDCS involving the sham condition will provide strong evidence of effects of tDCS. Currently systematic reviews that target randomized controlled trials and repeated measures studies on effects of anodal cerebellar tDCS on patients’ movements are lacking. This type of systematic reviews will show current evidence of behavioral studies to scientists and frontline practitioners for designing large-scale studies and making clinical decisions. In addition, protocols summarized in this type of systematic reviews will serve as a reference for healthcare providers’ consideration when anodal cerebellar tDCS is used as a clinical therapeutic modality. It should be noted that although there have been review articles regarding tDCS and cerebellar ataxias [18,21,22], the articles did not focus on anodal cerebellar tDCS and on studies adopting rigorous research designs (including the aforementioned randomized sham-controlled trials and repeated measures studies) or did not provide a systematic review.

The current systematic review addressed the following research questions: (1) Did anodal cerebellar tDCS improve movements in patients with cerebellar ataxias? and (2) What were the protocols of anodal cerebellar tDCS that effectively improved movements in patients with cerebellar ataxias? This systematic review only selected randomized sham-controlled trials and repeated measures studies with the random order of the active stimulation and the sham stimulation. Considering the purpose of this review was to investigate effects of cerebellar tDCS on various movements in patients with cerebellar ataxias, we did not restrict types of movements that were included in this review.

## 2. Methods

### 2.1. Inclusion and Exclusion Criteria

This systematic review followed the guidelines of Preferred Reporting Items for Systematic Reviews and Meta-Analyses (PRISMA). Studies that satisfied the following criteria were selected: (1) a topic examining effects of anodal cerebellar tDCS on movements in patients with cerebellar ataxias; (2) randomized sham-controlled trials or repeated measures studies with the random order of the active stimulation and the sham stimulation; (3) English publications; and (4) provision of the full text. Anodal cerebellar tDCS was defined as the tDCS with the anode being placed over any parts of the cerebellum and the cathode being placed over the other head region or the body of the patient.

Exclusion criteria were (1) conference proceedings, considering no peer review process in general; and (2) protocols, considering lack of experimental results.

### 2.2. Searching Strategies

The databases we searched were Web of Science, MEDLINE, PsycINFO, CINAHL, EMBASE, Cochrane Library, and EBSCOhost. The strategies used for searching in search fields of titles and abstracts of the databases were (1) “transcranial direct current stimulat*” and cerebell* and ataxi*; (2) tDCS and cerebell* and ataxi*; (3) “transcranial stimulat*” and cerebell* and ataxi*; (4) “direct current” and cerebell* and ataxi*; and (5) “current stimulat*” and cerebell* and ataxi*. The last search date for all databases was July 9, 2020. In order to further confirm whether articles extracted from the databases met the inclusion and exclusion criteria, two senior and experienced authors carefully read titles, abstracts, and the main text of articles and did the screening independently. They further discussed discrepancies in screening results to reach a consensus.

### 2.3. Qualitative Assessment

Methodological quality of the selected studies was assessed using the Physiotherapy Evidence Database (PEDro) scale [23,24], which consists of one item for assessing external validity, eight items for assessing internal validity, and two items for assessing statistical reporting. Each item was rated zero (not clearly satisfy) or one (clearly satisfy). The total score of the PEDro scale, which was the sum of scores of the internal validity items and the statistical reporting items, ranged from zero to ten. A total score below four, that of four to five, that of six to eight, and that of nine to ten were considered poor, fair, good, and excellent methodological quality respectively [24]. One study was rated by two raters separately. If the two raters gave different ratings to any PEDro item, a third rater was consulted. It has been reported [24] that the PEDro scale has satisfactory inter-rater reliability and construct validity. We chose the PEDro scale to rate the studies selected in this systematic review because this rating scale has good reliability and validity, can be used to comprehensively assess methodological quality of research, and has been applied to rating quality of medical studies [23,24].

## 3. Results

**Figure 1:**
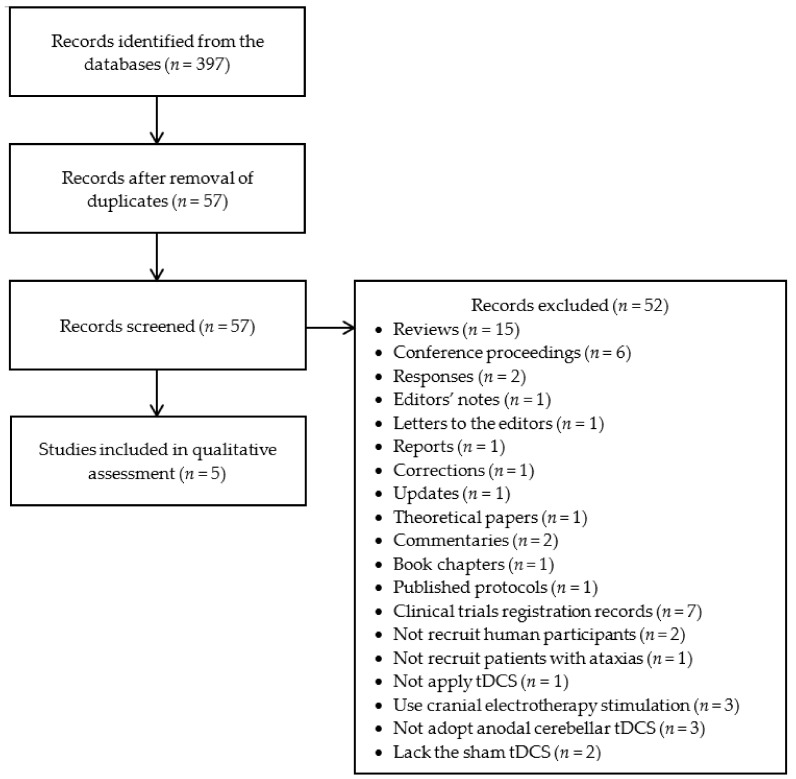
The flow diagram of study selection. tDCS = transcranial direct current stimulation.

A total of 397 records were identified from the databases and reduced to 57 records after removal of duplicates (Figure 1). Among the 57 records, 52 records were further excluded: 40 records were reviews [6,16,18,19,22,25,26,27,28,29,30,31,32,33,34], conference proceedings [35,36,37,38,39,40], responses [41,42], editors’ notes [43], letters to the editors [44], reports [45], corrections [46], updates [47], theoretical papers [48], commentaries [49,50], book chapters [51], published protocols [52], or clinical trials registration records [53,54,55,56,57,58,59]; two studies did not recruit human participants [60,61]; one study did not recruit patients with cerebellar ataxias [62]; one study did not apply tDCS [63]; three studies used cranial electrotherapy stimulation, CES [64,65,66]; three studies did not adopt anodal cerebellar tDCS [67,68,69]; and two studies lacked the sham tDCS [70,71]. In conclusion, a total of five papers were included in this systematic review [11,12,72,73,74]. Characteristics of these five studies are summarized in Table 1.

### 3.1. Study Designs of the Selected Studies

Among the five selected studies, four [11,72,73,74] were repeated measures studies; one [12] was a randomized controlled trial. For repeated measures studies providing the single active stimulation in the protocol [72,74], the washout period was from one week to two weeks. For repeated measures studies providing multiple active stimulation in the protocol [11,73], the washout period was from one month to three months.

### 3.2. Participants Recruited in the Selected Studies

The number of patients with cerebellar ataxias in each selected study [11,12,72,73,74] ranged from six to 20. Adult patients were targeted in four studies [11,12,72,74] (mean age: from 49.8 to 55.2 years in the studies); children with ataxias were targeted in one study [73] (mean age: 7.2 years). The percentage of female patients in each selected study [11,12,72,73,74] ranged from 40% to 58%. The four studies targeting adult patients [11,12,72,74] recruited 33 patients with spinocerebellar ataxias, 18 patients with sporadic adult-onset ataxias, 16 patients with cerebellar variants of the multiple system atrophy cohort, four patients with autosomal dominant cerebellar ataxias, three patients with ataxias with oculomotor apraxia, three patients with Friedreich’s ataxia, two patients with Fragile-X-associated tremor/ataxia syndrome, and one patient with cerebellitis. The study targeting children [73] recruited a total of six children with ataxic cerebral palsy. For adult patients [11,12,72,74], the mean disease duration ranged from 12.9 to 14.1 years; mean onset age ranged from 35.8 to 41.7 years.

### 3.3. Effects of Anodal Cerebellar tDCS on Movements

The nine-hole peg test, the eight-meter walking time, the timed up and go test, pediatric balance scale, and the pediatric evaluation of disability inventory were used to assess finger dexterity [11,12,72], gait speed [11,12,72], mobility [73], balance [73], and ability regarding self-care, mobility, and social function [73] respectively.

Anodal cerebellar tDCS improved finger dexterity in patients with cerebellar ataxias right after the stimulation protocols [11,12,72] and at one-month follow-up [11,12], but showed unstable positive effects on finger dexterity at three-month follow-up across studies [11,12]. Effects of anodal cerebellar tDCS on gait speed in patients with cerebellar ataxias were inconsistent. Two studies showed that anodal cerebellar tDCS improved gait speed right after the stimulation protocols [11,72], at one-month follow-up [11], and at three-month follow-up [11]. However, one study reported that anodal cerebellar tDCS showed no effects on gait speed right after the stimulation protocols, at one-month follow-up, and at three-month follow-up [12].

Anodal cerebellar tDCS showed no effects on mobility in patients with cerebellar ataxias right after the stimulation protocols, at one-month follow-up, and at three-month follow-up [73]. Similarly, anodal cerebellar tDCS showed no effects on balance and ability regarding self-care, mobility, and social function in patients with cerebellar ataxias right after the stimulation protocols and at three-month follow-up [73], although positive effects were reported at one-month follow-up [73].

Several movement parameters, including oscillations of the center of pressure, average movement time, average movement speed, perpendicular velocity, aiming errors, and the learning index, were calculated in studies using instrumental measures [73,74]. Anodal cerebellar tDCS showed positive effects on oscillations of the center of pressure in patients with cerebellar ataxias right after the stimulation protocols, at one-month follow-up, and at three-month follow-up [73]. However, anodal cerebellar tDCS showed no effects on the remaining instrumental measurements in patients with cerebellar ataxias during the stimulation protocol [74].

### 3.4. Additional Information: Effects of Anodal Cerebellar tDCS on Disease Severity

Both of *the scale for the assessment and rating of ataxia* and *the international cooperative ataxia rating scale* were used to assess disease severity [11,12,72]. Anodal cerebellar tDCS decreased disease severity in patients with cerebellar ataxias right after the stimulation protocols [11,12,72], at one-month follow-up [11,12], and at three-month follow-up [11,12].

### 3.5. Additional Information: Effects of Anodal Cerebellar tDCS on Quality of Life

*The stroke and aphasia quality of life scale—39 items* and *the short-form health survey—36 items* were used to assess quality of life [11,12]. Anodal cerebellar tDCS improved quality of life in patients with cerebellar ataxias right after the stimulation protocols, at one-month follow-up, and at three-month follow-up when *the short-form health survey—36 items* was the measure of quality of life [11]. However, anodal cerebellar tDCS only improved quality of life in patients with cerebellar ataxias right after the stimulation protocols, but not at one-month follow-up and three-month follow-up when *the stroke and aphasia quality of life scale—39 items* was the measure of quality of life [12].

### 3.6. Protocols of Anodal Cerebellar tDCS

Among the five selected studies, four studies showed positive effects of anodal cerebellar tDCS on movements in patients [11,12,72,73]. The remaining one study showed no effects of anodal cerebellar tDCS on movements in patients [74].

#### 3.6.1. Protocols Used in the Studies Showing Positive Effects

The anode was placed over the cerebellum [11,12,72,73]. The cathode was placed over the right deltoid muscle [12,72], the spinal lumbar enlargement [11], or the central supraorbital region [73]. The size of electrode sponges was 7×5 cm^2^ [11,12,72,73]. The current intensity was 2 mA [11,12,72] or 1 mA [73]. The stimulation duration per session was 20 min [11,12,72,73]. The total number of active anodal cerebellar tDCS sessions ranged from one [72] to 10 [11,12,73].

#### 3.6.2. Protocols Used in the Study Showing No Effects

The anode was placed over the right cerebellar hemisphere [74]. The cathode was placed over the right buccinator muscle [74]. The size of electrode sponges was 25 cm^2^ [74]. The current intensity was 2 mA [74]. The stimulation duration per session was variant across patients (mean:1289 s; standard deviation: 150 s) [74]. The total number of active anodal cerebellar tDCS sessions was one [74].

### 3.7. Adverse Effects of Anodal Cerebellar tDCS

Three studies [11,72,74] did not report information concerning adverse effects of anodal cerebellar tDCS in patients with cerebellar ataxias. One study [12] reported that no adverse effects were found in patients. One study [73] reported that patients had a tolerable feeling of tingling in the initial minutes of tDCS, and reported that no moderate or severe adverse effects were found.

### 3.8. Qualitative Assessment

Scores of the PEDro scale for the selected studies [11,12,72,73,74] were summarized in Table 2. Three studies [11,12,74] were considered to be good (with a score of six to eight) and two [72,73] were considered to be excellent (with a score of nine to ten) in terms of methodological quality.

## 4. Discussion

### 4.1. Effects of Anodal Cerebellar tDCS on Movements in Patients

Among the five selected studies [11,12,72,73,74], four studies [11,12,72,73] have reported that anodal cerebellar tDCS improves movements in patients with cerebellar ataxias. The remaining one study [74] showed no effects of anodal cerebellar tDCS on movements in patients, which may be due to the design of the stimulation protocol. In the study [74], the stimulation duration per session varied across patients, which may lead to confounding influences of the stimulation duration on experimental results and thus explain the result of no significant effects of anodal cerebellar tDCS on movements in patients. Apart from this possible reason, it is found that the study showing no effects of the stimulation [74] measured movements during the stimulation, whereas the other four studies showing positive effects of the stimulation [11,12,72,73] assessed movements after the stimulation. The results of the five studies [11,12,72,73,74] may reflect that anodal cerebellar tDCS is effective in improving movements in patients after the completion of the stimulation protocol, regardless of single or multiple stimulation, but not during the stimulation. It would be valuable to compare “online effects” (during stimulation) and “aftereffects” (after completion of stimulation) of anodal cerebellar tDCS on movements in patients in future research in order to facilitate the development of the most effective tDCS protocol.

The selected papers [11,12,72] have consistently reported that anodal cerebellar tDCS decreases disease severity and improves finger dexterity in patients with cerebellar ataxias right after the stimulation protocols and at one-month follow-up. The selected papers [11,12] have also shown that anodal cerebellar tDCS improves quality of life in patients right after the stimulation protocols. However, evidence is incongruent regarding positive effects of anodal cerebellar tDCS on gait control [11,12,72] and balance [73] in patients, which may be due to heterogeneity of research participants and choices of measures. Indeed, earlier research [72] has reported that positive effects of anodal cerebellar tDCS on gait control are shown in patients with spinocerebellar ataxias (*n* = 8) but not in patients with cerebellar variants of the multiple system atrophy cohort (*n* = 6). This result may reflect a possibility that anodal cerebellar tDCS exerts differential effects on gait control among patients with different types of cerebellar ataxias. In the selected studies [11,12,72], considering the rarity of each type of cerebellar ataxias [2], patients with different types of cerebellar ataxias were recruited as research participants in order to increase the sample size. It is likely that different combinations of ataxia types in the studies [11,12,72] lead to inconsistent results of effects of anodal cerebellar tDCS on gait control. In addition, it is noted that effects of anodal cerebellar tDCS on balance were observed when balance (i.e., oscillations of the center of pressure) was assessed using an instrumental measure but not observed when balance was assessed using a rating scale in the same study [73]. Taking into account that instrumental measures are less susceptible to rater bias and more sensitive to movement changes than rating scales [75], future research may adopt equipment to sensitively assess patients’ balance improvement resulting from anodal cerebellar tDCS.

### 4.2. Protocols of Anodal Cerebellar tDCS

Protocols used in the studies showing positive effects [11,12,72,73] adopted sponges of 7 × 5 cm^2^, placed the anode over the whole cerebellum, and put the cathode over the right deltoid muscle, the spinal lumbar enlargement, or the central supraorbital region. In general, the current intensity was set at 2 mA [11,12,72], and the stimulation duration per session was 20 min [11,12,72,73]. For the studies providing multiple sessions of anodal cerebellar tDCS [11,12,73], it is consistent that the total number of stimulation sessions was 10 with five sessions per week.

Considering the distance of each cerebellar lobe to the skull and the anode, it has been suggested [6] that the posterior lobe of the cerebellum is most likely to be stimulated by anodal cerebellar tDCS with the anode being placed over the whole cerebellum. Additionally, the current intensity of 2 mA is generally adopted in adult research participants in order to make the electric current strong enough to be able to reach the cerebellum and simultaneously prevent damage to brain tissue [6,76,77]. Notably, it has been reported [78] that Lobule VI and VIII of the posterior lobes of the cerebellum are involved in movement execution. Taken together, it seems reasonable that anodal cerebellar tDCS that could reach motor regions of the cerebellum [6,78] is effective in improving movements in patients with cerebellar ataxias. Future work will need to investigate influences of different current flow directions, related to the electrode placement, and different numbers and frequencies of stimulation sessions on effects of anodal cerebellar tDCS to benefit clinical application.

### 4.3. Clinical Implications

If clinical practitioners plan to provide intervention to improve finger dexterity and quality of life, or to ameliorate overall disease severity in patients with cerebellar ataxias, they may consider using anodal cerebellar tDCS. However, if improving balance or gait performance is the treatment goal, clinical practitioners should be cautious about using anodal cerebellar tDCS considering inconsistent research results regarding this effect.

When clinical practitioners apply anodal cerebellar tDCS to movement therapy for cerebellar ataxias, the suggested protocol may be using sponges of 7 × 5 cm^2^, placing the anode over the whole cerebellum, setting the current intensity at 2 mA, setting the stimulation duration per session at 20 min, and providing a total of 10 stimulation sessions with five sessions per week.

### 4.4. Limitations of This Systematic Review

The major limitation is that this systematic review included heterogeneous ataxia patients, which led to difficulty judging whether all types of ataxia patients could benefit from receiving anodal cerebellar tDCS. Nevertheless, it is very difficult for this systematic review to only focus on one specific type of ataxia patients to review effects of anodal cerebellar tDCS because few of this type of studies could be found. Due to rarity of each type of cerebellar ataxias, randomized controlled trials and repeated measures studies on cerebellar ataxias mainly recruited different types of ataxia patients in one study in order to secure enough sample size. Therefore, heterogeneity of ataxia participants is a common situation in ataxia studies. Another limitation is that we were unable to further conduct a meta-analysis given that varying outcome measures were used in the reviewed studies. In the future, with accumulated studies examining effects of anodal cerebellar tDCS on patients’ movements and adopting similar outcome measures, relevant meta-analyses will become feasible.

## 5. Conclusions

After systematically reviewing the literature, we have preliminary evidence to suggest that anodal cerebellar tDCS decreases disease severity and improves finger dexterity and quality of life in patients with cerebellar ataxias. The protocols of anodal cerebellar tDCS that effectively improved movements in patients commonly adopted sponges of 7 × 5 cm^2^, placed the anode over the whole cerebellum, set the current intensity at 2 mA, set the stimulation duration per session at 20 min, and provided a total of 10 stimulation sessions with five sessions per week. The results of this systematic review form a base when clinical practitioners consider applying anodal cerebellar tDCS to improving movements in patients with cerebellar ataxias. Future large-scale research needs to validate effectiveness of anodal cerebellar tDCS in homogeneous groups of patients, explore the optimal design of the stimulation protocol, and investigate responses in neural pathways to anodal cerebellar tDCS.

## Figures and Tables

**Table 1:** Characteristics of the selected studies.

Studies	Study Design	Ataxia Patients							Protocols of the Active tDCS ^c^						OutcomeMeasures andResults ^d^
		*n*	Age ^a^ (years)	Sex ^b^	Types	Disease Duration ^a^ (years)	Age at Onset ^a^ (years)		Anode	Cathode	Size of Electrode Sponges (cm^2^)	Intensity (mA)	Duration per Session (min)	No. of Sessions	
Benussi et al., 2015 [72]	Repeated measures (blinded tDCS providers, patients, and assessors)	19	53.8 ± 18.4	11F; 8M	1×SCA 1; 5×SCA 2; 2×SCA 38; 1×Friedreich’s ataxia; 1×AOA type 2; 6×MSA-C; 1×FXTAS; 2×SAOA	13.2 ± 14.7	40.6 ± 20.1		Cerebellum	Right deltoid muscle	7 × 5	2	20	Total: 1. Washout period ^e^: At least 1 week	After tDCS SARA: + ICARS: + 9HPT ^f^: + 8MW: +
Benussi et al., 2017 [12]	RCT (blinded patients and assessors)	20	Sham: 49.8 ± 16.7; Active: 55.2 ± 18.2	10F; 10M	5×SCA 2; 1×SCA 14; 2×SCA 38; 1×Friedreich's ataxia; 1×AOA type 2; 4×MSA-C; 1×FXTAS; 5×SAOA	Sham: 14.0 ± 12.9; Active: 13.8 ± 8.6	Sham: 35.8 ± 20.6; Active: 41.4 ± 20.9		Cerebellum	Right deltoid muscle	7 × 5	2	20	Total: 10. Per day: 1. Per week: 5	After tDCS SARA: + ICARS: + 9HPT-D: # 9HPT-nD: + 8MW: # SAQOL: + 1-month FU SARA: + ICARS: + 9HPT-D: + 9HPT-nD: + 8MW: # SAQOL: # 3-month FU SARA: + ICARS: + 9HPT-D: # 9HPT-nD: # 8MW: # SAQOL: #
Benussi et al., 2018 [11]	Repeated measures (blinded patients and assessors)	21 ^g^	54.6 ± 14.5	10F; 10M	7×SCA 2; 1×SCA 14; 1×SCA 38; 1×Friedreich's ataxia; 1×AOA type 2; 6×MSA-C; 4×SAOA	12.9 ± 12.6	41.7 ± 19.5		Cerebellum	Spinal lumbar enlargement	7 × 5	2	20	Total: 10. Per day: 1. Per week: 5. Washout period ^e^: 3 months	After tDCS SARA: + ICARS: + 9HPT-D: + 9HPT-nD: + 8MW: + SF: + 1-month FU SARA: + ICARS: + 9HPT-D: + 9HPT-nD: + 8MW: + SF: + 3-month FU SARA: + ICARS: + 9HPT-D: + 9HPT-nD: + 8MW: + SF: +
Grecco et al., 2017 [73]	Repeated measures (blinded patients and assessors)	6	7.2 ± 2.1	3F; 3M	Ataxic cerebral palsy	No information	No information		Cerebellum	Central supraorbital region	7 × 5	1	20	Total: 10. Per day: Unclear. Per week: 5. Washout period ^e^: 1 month	After tDCS COP-EC: + COP-EO: # PBS: # TUGT: # PEDI: # 1-month FU COP-EC: + COP-EO: # PBS: + TUGT: # PEDI: + 3-month FU COP-EC: + COP-EO: # PBS: # TUGT: # PEDI: #
Hulst et al., 2017 [74]	Repeated measures (blinded patients and assessors)	20	53.7 ± 10.8	8F; 12M	5×SCA 6; 3×SCA 14; 7×SAOA; 1×Cerebellitis; 4×ADCA III	14.1 ± 7.3	No information		Right cerebellar hemisphere	Right buccinator muscle	25 (unclear length and width)	2	No fixed duration	Total: 1. Washout period ^e^: 1 week or 2 weeks	During tDCS AMT: # AMS: # PV: # AEA: # AEW: # LI: #

tDCS = Transcranial direct current stimulation; SCA = Spinocerebellar ataxias; AOA = Ataxias with oculomotor apraxia; MSA-C = Cerebellar variants of the multiple system atrophy cohort; FXTAS = Fragile-X-associated tremor/ataxia syndrome; SAOA = Sporadic adult-onset ataxias; SARA = The Scale for the Assessment and Rating of Ataxia; ICARS = The International Cooperative Ataxia Rating Scale; 9HPT = The Nine-Hole Peg Test (D: For the dominant hand; nD: For the non-dominant hand); 8MW = The Eight-Meter Walking Time; RCT = Randomized controlled trials; SAQOL = The Stroke and Aphasia Quality of Life Scale-39 items; SF = The Short-Form Health Survey-36 items; COP-EC = Oscillations of the center of pressure with eyes closed; COP-EO = Oscillations of the center of pressure with eyes open; PBS = Pediatric Balance Scale; TUGT = The Timed Up and Go Test; PEDI = The Pediatric Evaluation of Disability Inventory; ADCA III = Autosomal dominant cerebellar ataxias type III; AMT = Average movement time; AMS = Average movement speed; PV = Perpendicular velocity; AEA = Aiming errors-adaptation; AEW = Aiming errors-washout; LI = Learning index. ^a^ Mean ± SD. ^b^ F = Female; M = Male. ^c^ Protocols of the sham tDCS were equivalent to those of the active tDCS except the current intensity. ^d^ +: Positive effects of the anodal cerebellar tDCS at different time points compared with pretest; #: No effects of the anodal cerebellar tDCS at different time points compared with pretest; FU = Follow-up. ^e^ A washout period between the active anodal cerebellar tDCS (and its entire follow-up period, if applicable) and the sham anodal cerebellar tDCS (and its entire follow-up period, if applicable). ^f^ Unclear information regarding whether this was the right-hand result or the left-hand result. ^g^ 21 patients were enrolled; one patient dropped out. Therefore, 20 patients were included in analysis.

**Table 2:** Scores of the PEDro scale for each selected study.

Items of the PEDro Scale	Benussi et al., 2015 [72]	Benussi et al., 2017 [12]	Benussi et al., 2018 [11]	Grecco et al., 2017 [73]	Hulst et al., 2017 [74]
**External validity**					
1. Source and inclusion criteria of participants	Yes	Yes	Yes	Yes	No
**Internal validity**					
2. Random allocation	1	1	1	1	1
3. Concealed allocation	0	0	0	1	0
4. Similar baseline data between groups	1	1	1	1	0
5. Blinding of subjects	1	1	1	1	1
6. Blinding of ones who provide therapy	1	0	0	0	0
7. Blinding of assessors	1	1	1	1	1
8. Study completers are more than 85%	1	1	1	1	1
9. Intention to treat analysis	1	1	1	1	1
**S** **tatistical reporting**					
10. Comparisons between groups/conditions	1	1	1	1	1
11. Point measures and measures of variability	1	1	1	1	1
**Total score**	9	8	8	9	7

PEDro = Physiotherapy Evidence Database.

## Data Availability

Not applicable.
